# Rescue of Foot-and-Mouth Disease Viruses That Are Pathogenic for Cattle from Preserved Viral RNA Samples

**DOI:** 10.1371/journal.pone.0014621

**Published:** 2011-01-28

**Authors:** Graham J. Belsham, Syed M. Jamal, Kirsten Tjørnehøj, Anette Bøtner

**Affiliations:** 1 National Veterinary Institute, Technical University of Denmark, Lindholm, Kalvehave, Denmark; 2 National Veterinary Laboratory, Islamabad, Pakistan; 3 Department of Microbiology, Quaid-i-Azam University, Islamabad, Pakistan; University of Hong Kong, Hong Kong

## Abstract

**Background:**

Foot and mouth disease is an economically important disease of cloven-hoofed animals including cattle, sheep and pigs. It is caused by a picornavirus, foot-and-mouth disease virus (FMDV), which has a positive sense RNA genome which, when introduced into cells, can initiate virus replication.

**Principal Findings:**

A system has been developed to rescue infectious FMDV from RNA preparations generated from clinical samples obtained under experimental conditions and then applied to samples collected in the “field”. Clinical samples from suspect cases of foot-and-mouth disease (FMD) were obtained from within Pakistan and Afghanistan. The samples were treated to preserve the RNA and then transported to National Veterinary Institute, Lindholm, Denmark. Following RNA extraction, FMDV RNA was quantified by real-time RT-PCR and samples containing significant levels of FMDV RNA were introduced into susceptible cells using electroporation. Progeny viruses were amplified in primary bovine thyroid cells and characterized using antigen ELISA and also by RT-PCR plus sequencing. FMD viruses of three different serotypes and multiple lineages have been successfully rescued from the RNA samples. Two of the rescued viruses (of serotype O and Asia 1) were inoculated into bull calves under high containment conditions. Acute clinical disease was observed in each case which spread rapidly from the inoculated calves to in-contact animals. Thus the rescued viruses were highly pathogenic. The availability of the rescued viruses enabled serotyping by antigen ELISA and facilitated genome sequencing.

**Conclusions:**

The procedure described here should improve the characterization of FMDVs circulating in countries where the disease is endemic and thus enhance disease control globally.

## Introduction

Foot-and-mouth disease (FMD) is one of the most economically important diseases of farm animals [1], [2]. The disease is widespread across the world especially in Africa and Asia. In addition, there are occasional incursions into countries which are normally free, e.g. within Japan (in 2000 and 2010) and the United Kingdom in 2001 (which resulted in spread to Ireland, France and the Netherlands) causing very high economic losses. The disease is caused by infection with foot-and-mouth disease virus (FMDV), the prototypic *Aphthovirus* within the family *Picornaviridae*. FMDV particles comprise a single copy of the positive-sense RNA genome (ca. 8300 nt in length) within a near spherical protein capsid of ca. 28 nm which contains 60 copies of 4 different structural proteins, 1A (VP4), 1B (VP2), 1C (VP3) and 1D (VP1) (for review, see [3]). The viral RNA is sufficient to initiate replication when introduced into the cytoplasm of cells (e.g., see [4], [5]), without any requirement for viral proteins Thus the capsid serves to protect the RNA when the virus is outside of cells and to facilitate delivery of the genome into the cytoplasm of cells in which replication occurs [3].

FMD can be controlled successfully by vaccination. However, there are seven distinct serotypes (O, A, C, Asia 1, SAT 1, SAT 2 and SAT 3) and many subtypes have also been described [2], [6]. Vaccination against one serotype produces little or no protection against other serotypes. Thus, it is essential that the correct vaccine is chosen for the control of each outbreak.

In countries lacking the infrastructure to identify rapidly the serotype of FMDV in samples, these can be transported to reference laboratories, e.g. the World Reference Laboratory at Pirbright, U.K., for characterization. However, this currently involves the transportation of samples containing infectious FMDV which represents a significant bio-security hazard and also incurs significant costs. Moreover, the virus samples have to be collected and stored under appropriate conditions, until submission, to allow virus to be isolated successfully in the receiving laboratories. In practice, this can be difficult when ambient temperatures are high and distances from points of collection to national laboratories are large and the infrastructure is poor. It is therefore desirable to have a system where samples from suspect clinical cases of FMD can be treated at the point of collection to inactivate the virus but preserve the RNA in a manner which allows the subsequent recovery of infectious virus within a contained laboratory environment.

The availability of commercial reagents which preserve the viral RNA, in conjunction with systems for the efficient rescue of picornaviruses from RNA (e.g. as used in [5]), has permitted the development of a protocol for the recovery of infectious FMDV from chemically treated virus samples. Indeed, a method for rescue of FMDV and classical swine fever virus from viral RNA stored in Trizol has been reported previously [7]. Related methodology has now been used to show that FMD viruses, which retain their pathogenicity, can be obtained from preserved virus preparations that were collected as clinical samples in one country and then transported to the National Veterinary Institute (DTU Vet), Lindholm, Denmark. This serves as a “proof of principle” for a system of efficient collection and preservation of FMDV samples in the field prior to comprehensive laboratory analysis of the rescued viruses.

## Materials and Methods

### Ethics statement

All animal work was approved and conducted according to the requirements of the Danish Animal Experiments Inspectorate (Licence no. 2008/561-1541).

### Preparation and quantification of FMDV RNA

An aliquot of bovine tongue epithelium suspension, stored at -70°C, (generated by Carolina Stenfeldt and Søren Alexandersen at DTU Vet, Lindholm) from a bull calf infected with FMDV (serotype O-UKG 34/2001) was used as a source of FMDV RNA obtained from clinical material. Nucleic acid was isolated using both robotic (MagNA Pure LC Total Nucleic Acid Isolation Kit) and manual (QIAamp®RNA Blood Mini Kit) protocols as described by the manufacturers and the RNA samples were eluted in 50 or 60 µl of water respectively.

FMDV RNA, within these RNA preparations, was quantified using two different real time RT-PCR assays, that target either the 5′ untranslated region or the 3D coding region, as described previously [8], [9], [10]. On the basis of these initial assays, samples containing a level of FMDV RNA which were expected to yield, in this standard diagnostic assay, Ct values of 15, 20, 25 and 30 respectively were prepared and re-assayed. It should be noted that each increase in Ct value of 5 represents a 2^5^ ( = 32)-fold decrease in the level of FMDV RNA.

Clinical samples (over 1500) were collected in Pakistan and Afghanistan as part of an FAO Regional Project (GTFS/INT/907/ITA). Mouth swab samples were placed directly into RLT buffer (Qiagen) while epithelium tissue samples, from clinically diseased animals, were initially collected in phosphate buffered saline (PBS) and then transferred to *RNAlater* (Ambion) in a laboratory prior to transportation to DTU Vet, Lindholm. From these preserved samples, RNA was isolated and the level of FMDV RNA was quantified by real time RT-PCR as above. Samples prepared from epithelium samples typically had the highest amounts of FMDV RNA.

### Rescue of infectious FMDV from FMDV RNA

Trypsinized BHK cells (800 µl of 2×10^6^ cells/ml) in PBS were mixed with 7 µl of RNA in a cuvette (0.4 cm) and subjected to a single, square wave, electrical pulse (25 ms) of 240V using a BioRad Gene Pulser Xcell electroporation system. The treated cells were added to a monolayer of primary bovine thyroid (BTY) cells (in a 6 well plate) and incubated at 37°C for 2 days prior to harvesting by freezing at −70°C. An aliquot (200 µl) of the harvest was added to fresh BTY cells and incubated for a further 1–2 days prior to harvesting as above. Cells were examined on a daily basis to assess the generation of CPE. Stocks of the rescued viruses were grown in BTY cells in 25 cm^2^ flasks and were titrated as described below.

### Characterization of rescued FMD viruses

The titre of the rescued virus samples was determined from 5 replicate dilution series in BTY cells (96 well plates), CPE was scored after both 1 and 2 days and the titre determined at 3 days post infection as TCID_50_ [11].

A previously described FMDV antigen ELISA [12], [13] was used to identify the serotype of the rescued FMDV and to demonstrate the presence of the expected serotype of FMDV in vesicular fluid obtained from experimentally infected animals (see below).

Samples of the virus harvest were also used to generate RNA samples as described above and used in RT-PCR assays using appropriate primer sets to obtain overlapping cDNA fragments corresponding either to selected regions of the genome or, in some cases, to the near complete genome (lacking the extreme 5′ untranslated region on the 5′ side of the poly(C) tract). DNA sequences of the amplicons were generated by Agowa (Germany). Sequence assembly was achieved using SeqMan Pro, sequence comparisons were performed using BLAST (www.ncbi.nlm.nih.gov) and the comparison of VP1 amino acid sequences was assessed using *MEGA* version 4 [14].

### Assessment of rescued virus pathogenicity in cattle

Two similar experiments were performed to determine the pathogenicity of two distinct rescued FMD viruses of different serotypes (O and Asia 1). In each case, two male calves (ca. 200 kg) were inoculated into the tongue, at two sites, with ca.10^7^ TCID_50_ (in total) of FMDV. Each inoculated animal was kept in contact with two other similar calves in pens within high containment animal accommodation and were monitored on a daily basis for signs of clinical disease (including elevated rectal temperature, drooling and appearance of vesicles in the mouth and on the feet) for a period of 10 days (unless euthanized prior to this time). Serum and mouth swab samples were collected before and daily after inoculation of the animals until euthanasia. RNA was then extracted from these samples and assayed for the presence of FMDV RNA by real time RT-PCR as above. The level of RNA detected in serum samples was converted to genomes per/µl by reference to a standard curve of reference RNA samples assayed in parallel (n.b. the level of genomes/µl will greatly exceed the level of virus detected by infectivity assays, commonly, for picornaviruses, a particle to pfu ratio of about 100 can be expected). The serum samples were also screened for the presence of anti-FMDV antibodies (using a 1∶10 dilution of serum) using serotype specific antibody ELISA systems (for O and Asia 1 as appropriate) and positive samples were then titrated to quantify the antibody content [15], [16].

## Results

### Rescue of virus from RNA isolated from FMDV-infected bovine tongue epithelium

**Figure 1:**
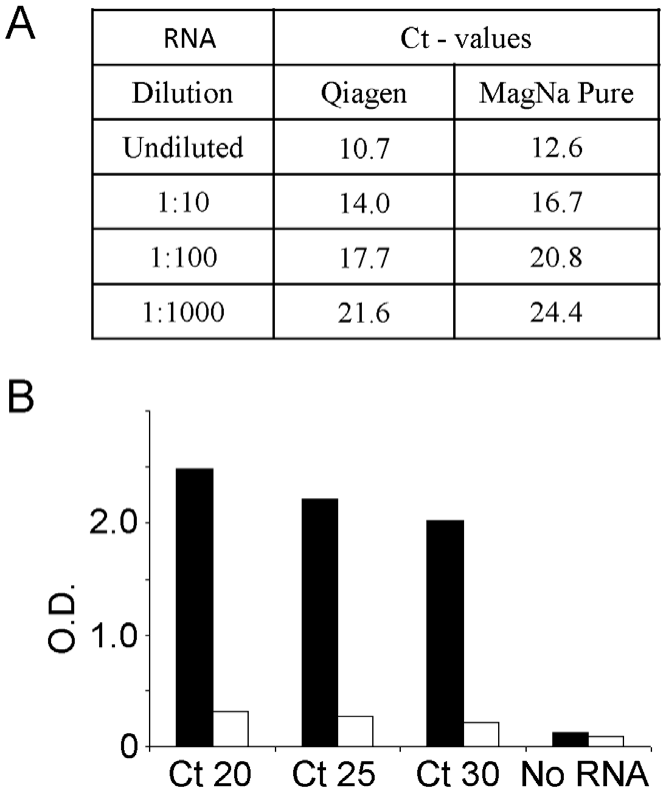
Rescue of serotype O FMDV following electroporation of BHK cells with RNA isolated from tongue epithelium suspension derived from a bovine infected with O UKG/35/2001 FMDV. Panel A. Quantitation of FMDV RNA by real-time RT-PCR following isolation by manual (Qiagen) or robotic (Roche MagNa Pure) systems. Panel B. RNA samples containing the indicated levels of FMDV RNA (as measured by real-time RT-PCR) were introduced into BHK cells by electroporation and rescued virus was propagated in BTY cells as described in materials and methods. The virus harvest (2^nd^ passage) was diluted (1∶50) and assayed in separate FMDV antigen ELISAs specific for serotype O (black bars) and A (open bars).

To optimize the procedure for the rescue of infectious FMDV from RNA preparations derived from clinical samples, initially RNA was isolated from bovine tongue epithelium suspension which was generated from cattle infected with serotype O FMDV (O-UKG 34/2001). The RNA was prepared both manually and by robot, then the FMDV RNA was quantified in each preparation using a standard diagnostic real-time RT-PCR assay [8]. The Ct values obtained for neat and a 10-fold dilution series of these samples are shown in Figure 1, panel A. As expected, a high level of FMDV RNA was present in both of these RNA preparations. A further dilution series was then made from the undiluted RNA preparation (QIAamp method) to generate samples with Ct values of 15, 20, 25 and 30 and the expected level of FMDV RNA was confirmed using the same assay (data not shown). Following optimisation of parameters for the introduction of FMDV RNA into BHK cells, a standard protocol was adopted as described in Material and Methods. Using this procedure, suspensions of BHK cells were electroporated in the presence of aliquots of the FMDV O UKG 34/2001 RNA dilution series (with Ct values of 20, 25 and 30). These cells were then added to a monolayer of primary bovine thyroid cells (BTY) and the harvested material was then passaged again on fresh BTY cells. On each day, the cells were examined for the appearance of CPE (see Table 1).

**Table 1:** Rescue of infectious FMDV from FMDV RNA.

FMDV RNA	1^st^ passage(BHK/BTY cells)		2^nd^ passage(BTY cells)	
Ct value	Day 1	Day 2	Day 1	Day 2
Ct 20	?	++	++	+++
Ct 25	?	neg	neg	+++
Ct 30	?	neg	neg	++

Different levels of FMDV RNA (expressed as the Ct value from the standard assay) were introduced into BHK cells by electroporation and added to monolayers of BTY cells. The cells were examined each day for virus production (as indicated by generation of CPE, ++ indicates partial or +++ complete cell lysis) For the second passage, 200 µl of virus harvest was added to a fresh monolayer of BTY cells. Neg indicates no CPE while the (?) indicates uncertainty due to the presence of many floating cells generated by the electroporation.

It should be noted that on day 1 following electroporation, it is difficult to score low levels of CPE due to the large number of dead and floating cells, however, when high levels of viral RNA were present in the samples, then CPE could be observed on day 2. Furthermore, even when the lowest level of RNA was used, the generation of CPE in the second passage was evident on day 2.

To determine that the CPE resulted from the rescue of virus from the electroporated FMDV RNA, a serotype specific antigen ELISA for FMDV was employed using the material harvested after the 2^nd^ passage. This assay has the feature that it has an absolute requirement for virus replication in order to generate the virus protein which is detected since only viral RNA was applied to the cells. The results are shown in Figure 1 (panel B). As expected from the source of the RNA (FMDV O UKG/34/2001) a strong signal was obtained for serotype O FMDV but no reactivity in a serotype A-specific assay was observed. Consistent with the generation of CPE that was obtained, a positive signal was observed in the serotype O specific antigen ELISA for each of these different RNA preparations.

### Rescue of FMDV from field samples preserved in RNAlater

Clinical samples (over 1500) were collected from cattle and buffalo from within Pakistan and Afghanistan. These materials included samples of bovine tongue epithelium from clinically diseased animals which were collected in PBS, transported back to the National Veterinary Laboratory in Pakistan or Afghanistan and then transferred to *RNAlater* to preserve the RNA and inactivate the virus. Following transportation to DTU Vet, Lindholm, total RNA was extracted from these samples and the presence of FMDV RNA was established and quantified using real time RT-PCR assays. With samples shown to contain FMDV RNA, conventional RT-PCR assays were used to amplify cDNA corresponding to the entire VP1 coding sequence and the amplicons (ca. 900 bp) were sequenced in order to identify the serotype of FMDV present within the clinical samples.

**Figure 2:**
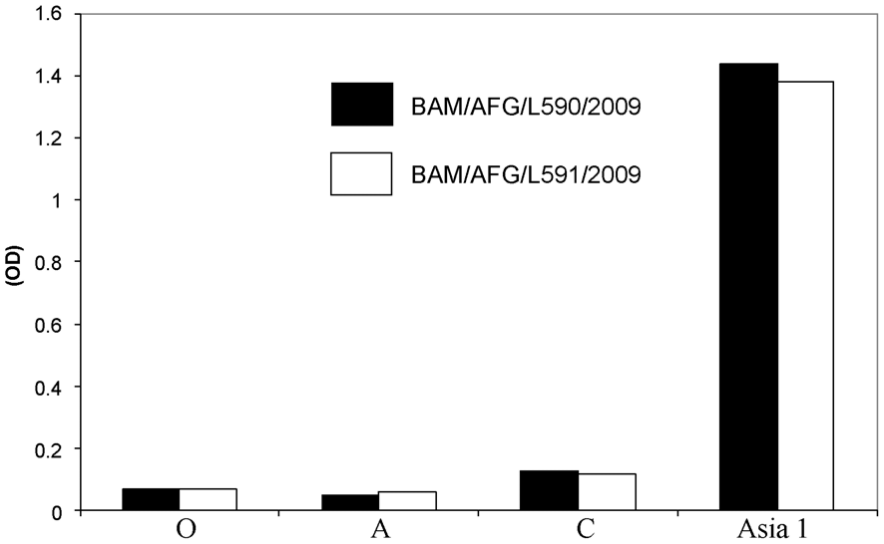
The rescued virus harvests Asia-1/BAM/AFG/L590/2009 (black bars, derived from RNA sample Asia-1/BAM/AFG/L1/2009) and Asia-1/BAM/AFG/L591/2009 (open bars, derived from RNA sample Asia-1/BAM/AFG/L2/2009) were diluted (1∶25) and analysed, in parallel, in separate FMDV antigen ELISAs specific for serotypes O, A, C and Asia 1 as indicated.

In an initial experiment, a collection of nine different RNA samples prepared from clinical samples obtained in Afghanistan and Pakistan and shown to contain high levels of FMDV RNA (by real-time RT-PCR) were selected to attempt virus rescue. From comparisons with other FMDV sequences (using BLAST) with the VP1 coding sequences of amplicons generated in conventional PCRs, it was known that these clinical samples contained FMDV RNA derived from either serotype A or Asia 1 viruses (see Table 2). Following introduction of these RNA samples into cells by electroporation and passaging in BTY cells, extensive CPE was apparent from two separate samples. Both of the RNA samples (Asia-1/BAM/AFG/L1/2009 and Asia-1/BAM/AFG/L2/2009) which were rescued back to virus (to produce the virus samples Asia-1/BAM/AFG/L590/2009 and Asia-1/BAM/AFG/L591/2009 respectively) had been prepared from bovine tongue epithelium collected in Afghanistan. In these RNA preparations, high levels of FMDV RNA were detected (Ct <20, see Table 2). Using the RNA prepared from the rescued viruses, sequencing of amplicons showed that the VP1 coding sequences were identical, in each case, to that generated from the input RNA. Furthermore, in an antigen ELISA, the rescued viruses were both serotyped as Asia 1, as expected from the sequence analysis, with no significant signal for serotypes O, A or C (Figure 2).

**Table 2:** Rescue of infectious FMDV from RNA samples isolated from bovine tongue epithelium from suspected cases of FMD in Afghanistan (AFG) and Pakistan (PAK).

Samples	Real time RT-PCR		Virus isolation (CPE)	
			1^st^ passage	2^nd^ passage
	5′-UTR (Ct)	3D (Ct)	BHK	BTY
Asia-1/BAM/AFG/L1/2009	19.4	16.1	Neg	+++
Asia-1/BAM/AFG/L2/2009	19.3	16.4	Neg	+++
Asia-1/BAM/AFG/L3/2009	21.8	19.9	Neg	Neg
A/SIN/PAK/L4/2008	26.6	20.2	Neg	Neg
Asia-1/SIN/PAK/L5/2008	21.4	19.3	Neg	Neg
A/SIN/PAK/L6/2008	24.2	18.2	Neg	Neg
A/SIN/PAK/L7/2008	27.6	19.3	Neg	Neg
Asia-1/SIN/PAK/L8/2008	21.8	19.8	Neg	Neg
A/SIN/PAK/L9/2008	29.0	21.1	Neg	Neg
O UKG/34/2001 (pos control)	20		Neg	+++
Neg contol	-	-	Neg	Neg

The presence of FMDV RNA was determined using real time RT-PCR assays which detect the 5′ UTR or the 3D coding sequence. The serotype of the virus from which the RNA was obtained was determined from VP1 coding sequence determination and comparison with other FMDV strains using BLAST. The success of virus rescue was determined by the presence of CPE (indicated by +++).

Subsequently, four other viruses have been rescued from epithelium samples collected in Pakistan and Afghanistan, each successful rescue was from RNA samples which had a Ct value of <20 (see Table 3). One of these virus samples was identified from sequencing of the VP1 coding region as containing serotype O FMDV (this rescued virus was designated O/ISL/PAK/L1573/2009 and was derived from RNA sample O/ISL/PAK/L1413/2009) while three others were serotype A; each of the latter belonged to the A IRN05 lineage but were distinct from the currently known sub-lineages, A IRN05^AFG-07^ and A IRN05^BAR-08^ (showing only 93 and 92% nucleotide sequence identity, respectively, within the VP1 coding region). As with the Asia 1 viruses, VP1 coding sequences determined from the initial RNA preparations and from the rescued serotype O and A viruses were identical except for a single nucleotide substitution. Thus, in total, some 850 bp have been sequenced from amplicons, including the complete VP1 coding sequence derived from the six RNA samples generated both before and after virus rescue, and only a single nucleotide discrepancy (out of over 10,000 nt examined) in one of the six rescued viruses has been detected.

**Table 3:** Rescue of multiple strains of FMDV from RNA samples isolated from bovine tongue epithelium from suspected cases of FMD in Afghanistan and Pakistan.

Virus sample and serotype(from VP1 sequencing)	Real time RT-PCR (Ct)(5′UTR)	VirusRescued	Serotype O Ag ELISA (OD)	Serotype AAg ELISA(OD)
A/KUN/AFG/L629/2009	16.6	-		
Asia-1/BAM/AFG/L640/2009	16.9	-		
Asia-1/BAM/AFG/L641/2009	16.4	-		
Asia-1/BAM/AFG/L643/2009	15.4	-		
O/SIN/PAK/L693/2009	21.8	-		
O/PUN/PAK/L1347/2008	20.2	-		
A/PUN/PAK/1354/2009	13.8	-		
A/PUN/PAK/1355/2009	21.0	-		
O/PUN/PAK/L1358/2008	17.9	-		
A/ISL/PAK/L1411/2009	19.2	-		
O/ISL/PAK/L1413/2009	19.0	+	**1.56**	0.09
A/BAL/AFG/L1430/2009	18.7	-		
A/SAR/AFG/L1435/2009	18.2	-		
A/HIR/AFG/L1483/2009	13.7	+	0.15	**1.53**
A/HIR/AFG/L1485/2009	14.4	+	0.03	* **0.31**
A/KAP/AFG/L1491/2009	15.1	-		
A/KUN/AFG/L1495/2009	16.3	+	0.21	**1.75**

The presence of FMDV RNA was determined using qRT-PCR assays, the serotype was determined from sequencing of amplicons including the VP1 coding sequence and then the RNA was introduced into BHK cells by electroporation as described in Material and Methods. When virus was successfully rescued, as determined by the generation of CPE (indicated by (+)), the harvested viruses (diluted 1∶25) were assayed for the presence of FMDV antigen using serotype specific ELISAs.

*Incomplete CPE was apparent in this sample.

Thus from 16 clinical samples, containing high levels of FMDV RNA (Ct value of <20) that were tested in the virus rescue system, some 6 different viruses have been rescued which are representatives of 3 different serotypes (O, A and Asia 1).

### Genome sequence analysis using rescued viruses

The availability of the rescued viruses greatly facilitated the process of generating near complete genome sequences. To obtain these sequences (ca. 8000 nt) for the rescued serotype O (O/ISL/PAK/L1573/2009) and Asia 1 (Asia-1/BAM/AFG/L590/2009) viruses, some 15 separate RT-PCR products were generated to provide overlapping cDNA fragments corresponding to all the genome sequence on the 3′ side of the poly(C) tract. These amplicons were sequenced in both directions and the sequences were then assembled and shown to include a single large open reading frame as expected. These sequences have been submitted to Genbank (accession numbers HQ113232 and HQ113233 for these type O and Asia 1 viruses respectively).

**Figure 3:**
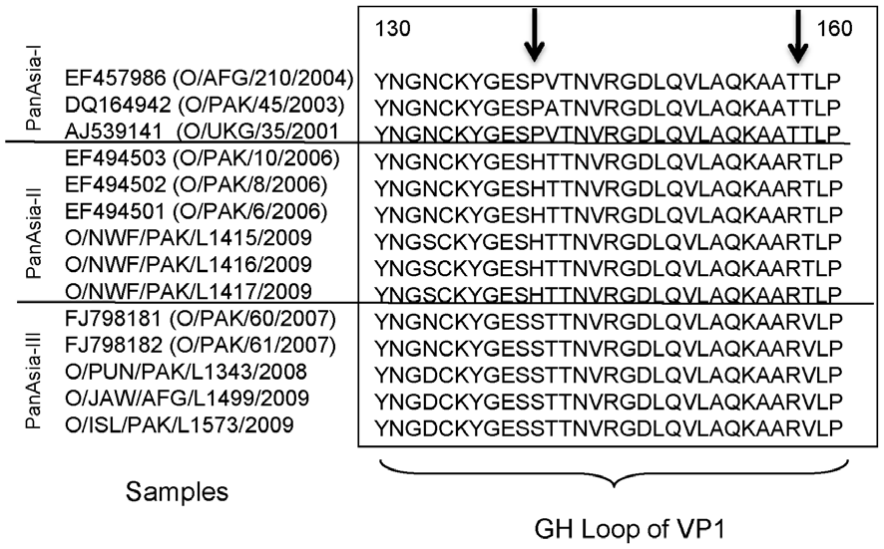
Diversity within VP1 amino acid sequences from PanAsia serotype O FMDVs including recent strains in circulation in Pakistan and Afghanistan. Amino acid sequences for the G-H loop portions of the VP1 capsid protein (which is important for FMDV antigenicity and receptor binding) were derived from reference PanAsia I and PanAsia II strains available in GenBank (accession numbers are indicated) and aligned with the derived VP1 sequences from current serotype O strains circulating in Pakistan and Afghanistan obtained in this study. The positions of shared amino acid substitutions are indicated by arrows.

The Asia-1/BAM/AFG/L590/2009 virus is most closely related (96–98% nt identity in VP1 coding sequences) to viruses causing disease outbreaks in 2003 in Tajikistan, Uzbekistan and Afghanistan, in 2004 in Kyrgyzstan and Pakistan and in 2005 in Hong Kong and this represents a reappearance of this serotype in this region. The serotype O virus (O/ISD/PAK/L1573/2009) is related to the Pan Asia II strains that have circulated widely in recent years. However the VP1 coding sequence is only about 94% identical to the representative PanAsia II viruses [e.g. O/Pak4/2006 (accession no. EF494501.1) and O/Pak6/2006 (accession no. EF494500.1)] and thus represents a new sub-lineage. This has been tentatively assigned as a PanAsia III lineage but this will probably be dependent on the survival and spread of this strain. Analysis of the near complete genome sequences of this virus and other more typical PanAsia II strains (which are still in circulation in Pakistan (e.g. O/NWF/PAK/L1417/2009)) indicated a sequence identity of about 95% across the entire genome. However, it is interesting to note that the deduced amino acid sequences of the VP1 proteins from the O/ISL/PAK/L1573/2009 virus and some other samples collected in Pakistan during 2009 differ from the Pan Asia II serotype O viruses which are still currently circulating there and include changes flanking the receptor binding motif (RGDLXXL) within residues 140–160 (G-H loop) which is an important antigenic site (see Figure 3). A fuller analysis of the different serotype O viruses currently circulating in Pakistan will be reported separately (Jamal et al., submitted for publication).

### Analysis of rescued FMDV pathogenicity in cattle

To test the pathogenicity of the rescued viruses, two experiments were performed using the serotype Asia 1 and serotype O viruses. In each case, two bull calves were inoculated into the tongue and each of these was then kept with two in-contact calves and all six animals were closely monitored over a period of 10 days during which time serum and mouth swab samples were collected. The presence of FMDV RNA in these samples was determined and the generation of anti-FMDV antibodies within the sera was assayed.

**Figure 4:**
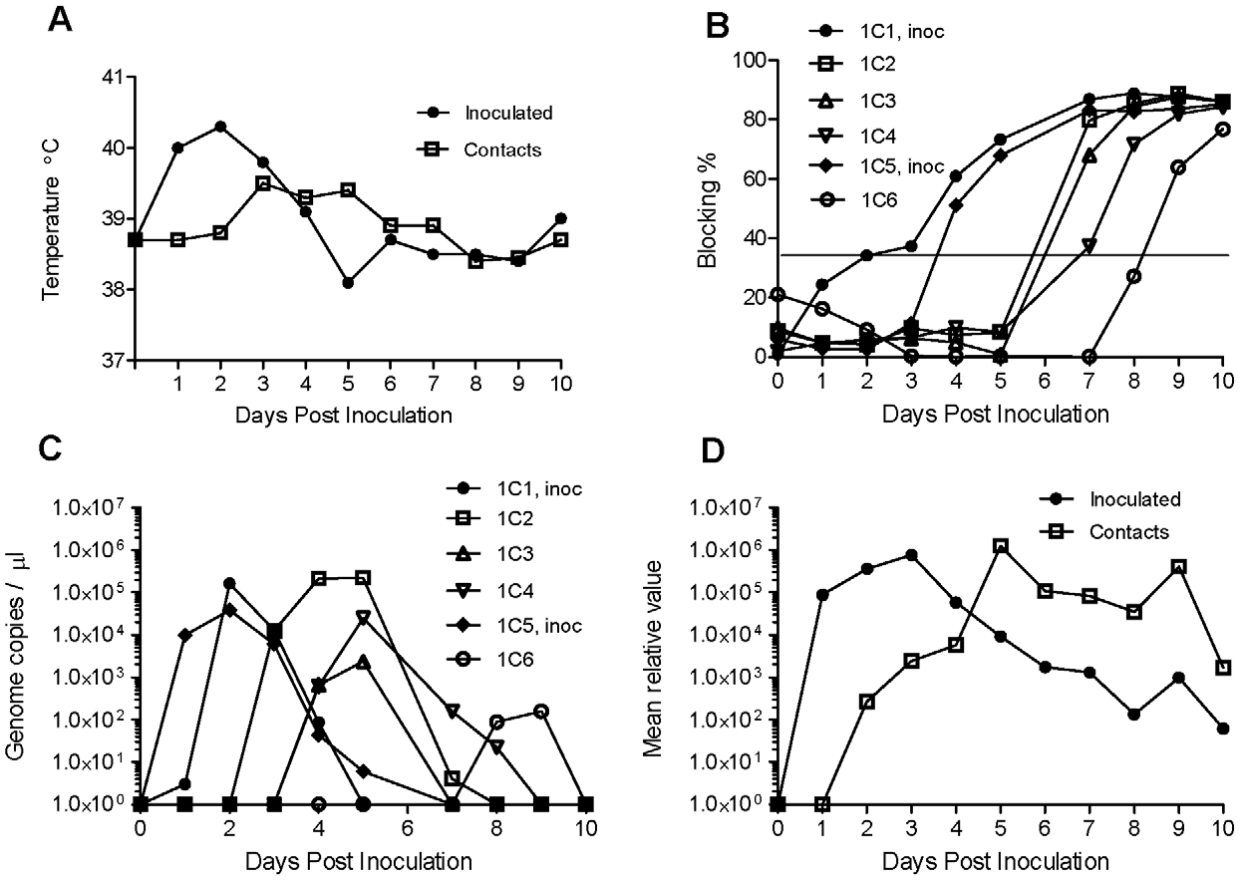
Inoculation and infection of bovine calves by the rescued FMDV Asia-1/BAM/AFG/L590/2009. Calves 1C1 and 1C5 were inoculated into the tongue with the rescued virus (10^7^ TCID_50_) and housed in contact with 4 control animals (in 2 separate pens containing calves 1C1-1C3 and 1C4-1C6 respectively). The calves were monitored for clinical signs (including temperature, note these are mean values for the animals within each group for clarity, panel A) and the presence of lesions in the mouth and on the feet). Oral fluid was collected as swab samples, in addition blood samples were obtained and the serum was assayed for the presence of anti-Asia 1 FMDV antibodies (panel B) in a screening assay using a single dilution of serum (1∶10). Positive samples (blocking >35%, which is the diagnostic cut off point and indicated by a horizontal line) were titrated (see Table 4). The level of FMDV RNA in serum samples was also determined by real-time PCR and expressed as the number of RNA copies/µl serum (panel C) and the presence of FMDV RNA in mouth swab samples (mean of the values determined for each group) is indicated in panel D. Note, due to differences in the amount of sample collected on a swab the level of FMDV RNA has not been converted to the number of genome copies but expressed in relative terms.

In the experiment using the rescued serotype Asia 1 virus (Asia-1/BAM/AFG/L590/2009), the two inoculated cattle exhibited an elevated temperature at 1 dpi and this remained high for another 2 days (Figure 4A). Vesicles appeared in both the interdigital space of the front feet and the coronary band of the rear feet on day 3 pi. In addition, vesicles were also observed from day 3 or 4 on both the upper and lower gingiva and both animals were drooling. These clinical signs of FMD continued for some days but visible healing was apparent from day 7 pi. The in-contact animals had a normal temperature until 3 days after the inoculation of the other cattle and then a more moderate elevation in temperature was observed which persisted for about 3 days (Figure 4A). Vesicles on the front and rear feet of these animals became apparent from day 4. In addition, vesicles were observed from days 4–5 on the tongue and on both the upper and lower gingiva which were also accompanied by drooling.

Sera collected from the animals were tested for antibodies against FMDV in a competition ELISA, initially using a single dilution of serum (Figure 4B). Both the inoculated cattle (1C1 and 1C5) had seroconverted to FMDV at 4 dpi and two of the four in-contact animals had clearly seroconverted by day 7 while the remaining two seroconverted on days 8 and 9. Sera which were shown to be positive in this assay were also titrated (see Table 4). The titres of antibodies against FMDV were still fairly low in two of the in-contact animals (1C4 and 1C6) at the termination of the experiment on day 10 but in each of the others a high titre (1∶160) was observed. Quantification of the FMDV RNA in the serum samples (Figure 4C) showed that viræmia occurred at days 1–4 in the inoculated animals while in the in-contact animals viræmia occurred between days 2–9 (but only for a period of up to 4 days in any one animal) and then subsided coincident with the induction of anti-FMDV antibodies in the serum. FMDV RNA was detected in mouth swab samples from day 1 in the inoculated animals and from day 2, initially at a rather lower level, in the in-contact animals (Figure 4D) and then persisted in both groups of animals, but at a declining level, for the rest of the experiment (until 10 dpi).

**Table 4:** Titration of anti-FMDV serotype Asia 1 antibodies induced following infection with rescued serotype Asia 1 FMDV.

	Calves					
dpi	1C1	1C2	1C3	1C4	1C5	1C6
-4	-	-	-	-	-	-
1	-	-	-	-	-	-
2	-	-	-	-	-	-
3	-	-	-	-	-	-
4	<10	-	-	-	-	-
5	10	-	-	-	<10	-
6	N.D.	N.D	N.D.	N.D.	N.D.	N.D.
7	160	20	<10	-	80	-
8	160	160	40	<10	160	-
9	160	160	80	40	160	<10
10	160	160	160	40	160	20

Values are reciprocals of the highest serum dilutions yielding positive results in the blocking ELISA on the indicated day post inoculation (dpi). A (-) indicates samples which were scored negative in the spot test (see Figure 4B) and N.D. indicates when samples were not collected. Calves 1C1 and 1C5 were inoculated with the rescued serotype Asia 1 FMDV (Asia-1/BAM/AFG/L590/2009) on day 0 and the other calves were maintained in contact with them.

**Figure 5:**
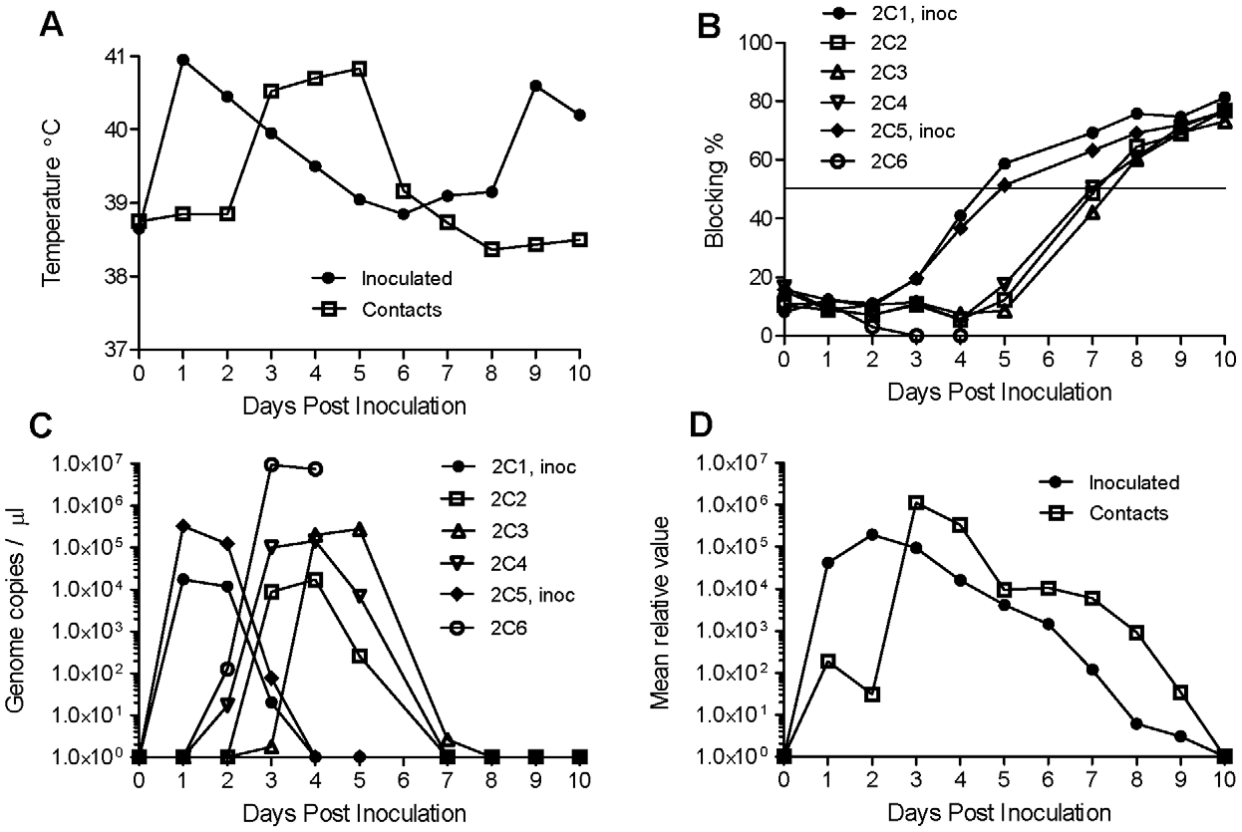
Inoculation and infection of bovine calves by rescued FMDV (O/ISL/PAK/L1573/2009). Calves 2C1 and 2C5 were inoculated into the tongue with the rescued virus (10^7^ TCID_50_) and housed in contact with 4 control animals (in 2 separate pens containing calves 2C1-2C3 and 2C4- 2C6 respectively). The calves were monitored for clinical signs (including temperature (mean values for the animals in each group are shown, panel A) and the presence of lesions in the mouth and on the feet). Note, on days 9 and 10 the inoculated calves were seen to be suffering from a secondary bacterial infection which presumably accounted for the second temperature peak. Oral fluid was collected as swab samples and blood samples were obtained and the serum was assayed for the presence of anti-O FMDV antibodies in a screening assay using a single dilution of serum (1∶10) (panel B). Positive samples (blocking >50%, which is the diagnostic cut off point and indicated by a horizontal line) were titrated (see Table 5). Note, calf 2C6 was euthanized at 4 dpi due to the severity of the clinical symptoms observed. The presence of FMDV RNA in serum samples was also determined by real-time PCR and expressed as the number of RNA copies/µl serum (panel C) and the presence of FMDV RNA in mouth swab samples (mean of the values determined for each group) is shown in panel D as in Figure 4.

Similarly, in the experiment using the rescued serotype O virus (O/ISL/PAK/L1573/2009), the two inoculated cattle exhibited a markedly elevated temperature at 1 dpi which gradually subsided over the next 3 days (Figure 5A). Vesicles appeared on the upper gingiva in both animals at 2 dpi and on the feet at 3 dpi. The in-contact animals had a normal temperature until 3 days after the challenge cattle were inoculated and then a significant elevation in temperature was observed which persisted for 3 days. All the animals developed vesicles in the mouth and on the feet by 4 dpi and due to the severity of lesions observed (severe damage to the tongue epithelium plus vesicles on all four feet) in one of the in-contact animals (2C6) it was euthanized on day 4.

Both the inoculated cattle (2C1 and 2C5) seroconverted to FMDV at 5 dpi and the three surviving in-contact animals seroconverted on day 8 (see Figure 5B). Not surprisingly, no antibody response was detectable in animal 2C6 at the time it was euthanized. It is apparent from Figure 5B that a rising level of anti-FMDV antibodies could be detected in the sera prior to their level reaching the diagnostic cut off point (this was also true in the experiment with the Asia 1 virus, see Figure 4B). Positive sera were titrated (see Table 5) and it was found that high levels of anti-FMDV antibodies were generated in the two inoculated animals and in one of the in-contact animals but the antibody titres were still moderate (1∶80) in the remaining two in-contact animals (2C2 and 2C3) at the termination of the experiment on day 10.

**Table 5:** Titration of anti-FMDV serotype O antibodies induced following infection with rescued serotype O FMDV.

	Calves					
dpi	2C1	2C2	2C3	2C4	2C5	2C6
0	-	-	-	-	-	-
1	-	-	-	-	-	-
2	-	-	-	-	-	-
3	-	-	-	-	-	-
4	-	-	-	-	-	-
5	20	-	-	-	10	N.D.
6	N.D.	N.D.	N.D.	N.D.	N.D.	N.D.
7	320	10	<10	10	160	N.D.
8	320	40	40	40	160	N.D.
9	320	80	40	80	80	N.D.
10	640	80	80	160	160	N.D.

Values are reciprocals of highest serum dilutions yielding positive results in the blocking ELISA on the indicated day post inoculation (dpi). A (-) indicates samples which were scored negative in the spot test (see Figure 5B) and N.D. indicates when samples were not collected (calf 2C6 was euthanized on day 4). Calves 2C1 and 2C5 were inoculated with serotype O FMDV (O/ISL/PAK/L1573/2009) on day 0 and the other calves were maintained in contact with them.

Quantification of the FMDV RNA in the serum samples (Figure 5C) showed the peak viræmia occurred at days 1 and 2 in the inoculated animals and then subsided as the anti-FMDV antibodies became detectable in the serum from day 3. In the in-contact animals, the peak viræmia occurred at days 3 to 5 post inoculation of the other animals and had significantly reduced by day 7 when anti-FMDV antibodies became detectable in the serum (Figure 5B). It is interesting to note that the highest level of viræmia was detected in one of these in-contact animals, calf 2C6, which was euthanized due to the severity of clinical disease. The level of FMDV RNA reached 7.4–9.3×10^9^ genomes/ml serum on days 3 and 4 which was about 30-times higher than observed in any other animal (see Figure 5C); the next highest level was 3.2×10^8^ genomes/ml observed in the inoculated calf 2C5 on day 1 post inoculation.

In this experiment, significant levels of FMDV RNA were also detected in mouth swab samples from day 1 in the inoculated animals and from day 3 in the in-contact animals which then gradually declined through to the completion of the experiment (see Figure 5D). It is apparent that the detection of viral RNA in the oral swabs was possible for a much longer period of time than the period of viræmia (see Figure 4 C, D and Figure 5 C, D).

## Discussion

In this study we have successfully demonstrated a process of rescuing infectious FMDV from RNA extracted from preserved clinical samples and the work confirms and significantly extends previous studies [7]. In this procedure, clinical samples were collected from suspected cases of FMD in Pakistan and Afghanistan and then treated to preserve the FMDV RNA. The samples were then transported to a high containment laboratory; viral RNA was isolated and then introduced back into cells, by electroporation, to rescue infectious FMDV. Importantly, we have shown that FMD viruses of three different serotypes (O, A and Asia 1 and also different sub-lineages of these serotypes) can be rescued from clinical samples collected in the field. Furthermore, it has been shown that the two rescued viruses which have been examined (each derived from epithelium samples from animals exhibiting clinical disease) were each highly pathogenic in cattle in Denmark. The fidelity of the rescue system was further supported by sequence analysis of RT-PCR derived amplicons corresponding to the VP1 coding sequence obtained directly from the initial clinical samples and also following virus rescue within cultured cells. Just 1 nt difference was detected within 10,000 nt of sequence information derived from samples tested before and after virus rescue. In principle it is possible that some sequence changes occurred elsewhere in the genome but clearly the rescued viruses retained the ability to replicate within cells in culture and also to cause disease in cattle. There seems no reason to believe that this procedure for obtaining infectious FMDV should be any less representative than standard virus isolation procedures. Availability of the rescued viruses facilitates the characterization of the circulating viruses including the identification of the serotype by antigen ELISA and also the near complete genome sequence determination. Furthermore, the rescued viruses are also available for antigenic profiling (e.g. using a panel of monoclonal antibodies) or virus neutralization assays to assess the efficacy of current vaccine strains to combat them.

Both the FMDV Asia 1 strain from Afghanistan and the serotype O strain (tentatively assigned as belonging to a new PanAsia III sub-lineage) from Pakistan obtained through this virus rescue procedure proved to be capable of initiating severe clinical disease when inoculated into the tongues of calves kept within high containment animal accommodation. Furthermore, the initial infections rapidly spread to in-contact animals and classical symptoms of FMD became apparent in each of the animals with fever and multiple lesions in the mouth and on the feet. The profile of viræmia (lasting 3–4 days) and the induction of an anti-FMDV immune response detectable from 4–5 days post inoculation were fairly typical (see [1]). It was apparent that a very high level of viræmia (ca. 7–9×10^9^ genomes/ml of serum) was detected with the serotype O virus in an in-contact animal which showed particularly severe clinical signs of disease (and was therefore euthanized at an early stage of the experiment). Due to the high particle to pfu ratio (ca. 100) known for FMDV, and other picornaviruses, it is likely this level of viræmia is roughly equivalent to the high level (ca. 10^7.8^ TCID_50_/ml) described for the serotype C Noville strain of FMDV in cattle (see [1]).

A feature of the virus rescue system described here is that virus amplification is achieved by infection of primary bovine cells (BTY). This system can be expected to maintain the “field” characteristics of the viruses and not introduce specific adaptations to cell culture within the capsid region which are frequently seen in BHK cells for example. The initial introduction of the viral RNA into BHK cells by electroporation was required due to the large number of cells used for this process and is not expected to induce adaptations, at least within the capsid region, since no capsid functions were required to initiate the infection cycle within these cells. The marked pathogenicity of the rescued viruses studied here clearly shows that this characteristic of these viruses was not significantly diminished in this procedure. There has been a recent description [17] of a goat epithelial cell line which is highly sensitive to FMDV and we have attempted to introduce FMDV RNA directly into these cells but have been unsuccessful, the cells either died or did not take up the RNA. However, it may well be possible to replace the primary BTY cells by this goat cell line for the propagation of the rescued virus.

Using RNA samples generated directly from clinical material within our laboratory, it has been possible to rescue infectious FMDV from samples containing quite low levels of viral RNA (Ct value of 30). However, using RNA samples derived from clinical samples collected in Pakistan and Afghanistan and then transported to Denmark, we have found that only samples containing a much higher level of FMDV RNA (Ct value of about 20) have been successfully rescued, this represents about a1000-fold higher level of viral RNA. It should be noted that even a single break in the RNA strand (ca. 8300 nt) is sufficient to destroy the infectivity of the RNA whereas it will have rather little effect on the apparent level of FMDV RNA detected in the real time RT-PCR assays as these target small regions of the genome (ca. 100–150 nt). Furthermore, the epithelium samples used here were transported to regional laboratories before the RNA preservation was performed. Direct placement of such samples into an RNA preservative at the point of collection can be expected to improve the efficiency of virus rescue. Clearly, if virus can be rescued from swab samples collected directly into an RNA preservative then this will be beneficial, on the basis of simplicity, and thus attempts to achieve this are in progress.

When outbreaks of FMDV occur, it is important for national authorities to be informed of the characteristics of the strain to allow selection of appropriate vaccines. To obtain detailed information about the properties of an outbreak strain, analysis of the live virus is required. The identification of serotype can be achieved by antigen ELISA if virus is available; however the sensitivity of these assays is relatively low so the ability to grow the virus can be important. Serotype identification can also be achieved by sequencing of amplicons corresponding to the VP1 (1D) coding sequence. Such assays can be performed directly on RNA obtained from clinical material but the analyses are greatly facilitated by the ability to grow the virus and hence obtain RNA preparations containing high levels of FMDV RNA. The pathway of infections within an outbreak can be traced using full-length genome sequence analysis [18], [19]. This is much more readily achievable with viruses that can be amplified in cell culture. Finally, an assessment of the ability of antisera generated by vaccines to neutralize outbreak strains is clearly desirable and this also requires the availability of infectious virus. It is apparent that this system to rescue FMDV from preserved RNA samples should be a useful step forward in attempts to monitor FMDV circulation within countries where the disease is endemic and hence aid attempts to control it globally.
